# Changes in lower limb muscle synchronisation during walking on high-heeled shoes

**DOI:** 10.1049/htl.2018.5032

**Published:** 2018-11-14

**Authors:** Manisha Pratihast, Ahmed Al-Ani, Rifai Chai, Steven Su, Ganesh Naik

**Affiliations:** 1 Centre for Health Technologies, Faculty of Engineering & IT, University of Technology Sydney, 15, Broadway Ultimo Sydney, New South Wales 2007, Australia; 2 Department of Telecommunications, Electrical, Robotics and Biomedical Engineering, Faculty of Science, Engineering & Technology, Swinburne University of Technology, PO Box 218, Hawthorn, Vic 3122, Australia; 3 Biomedical Engineering and Neuroscience Research Group, MARCS Institute, Western Sydney University, Kings Wood, 2747, New South Wales 2007, Australia

**Keywords:** muscle, footwear, biomechanics, medical signal processing, neurophysiology, gait analysis, electromyography, lower limb muscle synchronisation, high-heeled shoes, walking, average coherence values, RF-VL, RF-VM, RF-ST muscles, muscle pairs, increased beta band coherence, neuromuscular disorders, heel heights, muscle pair synchronisation, size 4.0 cm, size 6.0 cm, size 10.0 cm, frequency 15.0 Hz to 30.0 Hz

## Abstract

The goal of this research was to investigate the effect of wearing high-heeled shoes (HHS) on lower limb muscle synchronisation during walking, using beta band (15–30 Hz) coherence analysis. Fifteen females with no previous neuromuscular disorders volunteered in this study. Surface electromyography in frequency domain was studied from rectus femoris (RF), vastus lateralis (VL), vastus medialis (VM) and semitendinosus (ST) muscles during walking by subjects wearing HHS of three different heel heights (low – 4 cm, medium – 6 cm and high – 10 cm). Average coherence values were calculated for RF-VL, RF-VM and RF-ST muscles in beta band to analyse muscle pair synchronisation. In this study, significant increase in beta band coherence was found in all three muscle pairs during walking on HHS of different heel heights (*p*<0.05). Increased beta band coherence obtained from this study suggested that walking on HHS demands higher muscle pair synchronisation, to maintain stability around the knee joint.

## 1 Introduction

Walking in high-heeled shoes (HHS) is a very common trend among women, as it is believed that HHS makes women more attractive [1, 2]. Over the past several years, researchers have shown that walking on HHS may affect body posture and lower extremities of women [3–5] and that wearing them for longer duration may affect neuromechanics and kinematics of the lower limbs [6, 7]. Wearing HHS during walking changes movement around joint through the lower extremities. Knee movement pattern changes due to variation in muscle synchronisation pattern from low heeled to high heeled shoes during walking [7].

In a neural system, synchronisation of motor neurons is considered essential for any muscle activity. Different neural oscillations help muscles to be synchronised to perform day-to-day task and hence these neural oscillations in human beings have been a focus of research for several years [8]. Quadriceps and hamstring muscle synchronisation play an important role in human walking [9]. During this synchronisation, common neural inputs are sent to these muscles from the central nervous system (CNS). Common neural oscillations from the CNS are observed in beta band (15–30 Hz) [10] during human walking. According to previous research [8, 11], the common drive is present only in a muscle pair that has similar functions to maintain stability around a common joint during walking. Common neural inputs to muscle pairs can be examined using electromyography (EMG)-EMG coherence analysis during the walking task [8, 12, 13], which provides a synchronisation pattern of muscle pairs in the frequency domain.

EMG-EMG coherence analysis is a most popular approach to assess the common drive since it is easy to obtain, requiring only the recorded EMG signals from muscles without the need to perturb or stimulate the nervous system [14–17]. Previous studies have indicated that muscle fatigue increases beta band coherence [10] which is related to greater motor unit synchronisation. Beta band coherence can be expected to increase between motor units receiving common input that could be muscle pairs around the common joint and perform similar actions [18].

Walking on high heeled shoes demands lower limb muscle synchronisation changes to maintain stability around the knee. Along the sagittal plane, knee flexion is greater in high-heeled gait when compared to low-heeled gait. Hip flexion is also relatively less in magnitude when walking in high heels [7]. Changes in muscle activation pattern during walking in HHS have not been well documented by medical science or biomedical engineering research.

Therefore, the purpose of this study was to identify changes in lower limb muscle synchronisation of women when walking in the different height of HHS (low – 4 cm, medium – 6 cm, high – 10 cm). These changes were analysed using beta band coherence analysis, which quantified the synchronisation between muscle pairs.

## 2 Materials and methods

The University of Technology Sydney, Sydney (UTS) Human Research Ethics Committee approved the experimental protocol (Ethics details: UTS HREC 2013000728) for this study. Fifteen healthy young women participated in this study [age: 24.2 ± 1.5 years and body mass index:18.9 ± 1]. Subjects having any neurological/orthopaedic disorder, pregnancy, amputations and arthritis were excluded from the study (experiment). An information sheet was given, and all the participants signed a consent form before the experiment.

The initial assessment was carried out in the control shoes, so far worn by the subjects, to establish baseline surface electromyography information. Since the main aim of the study was to identify changes in lower limb muscle synchronisation of women when walking in the different HHS, the baseline information was not considered for further analysis. Afterwards, all subjects wore pointed high heeled shoes of 4, 6 and 10 cm heights, with almost similar shape (surface area 1 cm^2^) and style (stiletto).

Bipolar surface EMG sensors (silver-silver triode with a fixed inter-electrode distance of 2 cm and a diameter of 1 cm, Thought Technology, Montreal, Quebec, Canada) were placed on vastus medialis (VM), rectus femoris (RF), vastus lateralis (VL) and semitendinosus (ST) muscles that are mainly responsible for walking task [9]. The placement of electrodes was configured according to SENIAM guidelines [19]. The skin was carefully shaved and rinsed with pure alcohol, and the electrodes were connected to custom-built preamplifiers (input impedance, 80 MΩ) that were taped to the skin. The EMG signals were acquired at 2048 samples/s from different muscles. Afterwards, they were filtered between 10 and 450 Hz using fourth-order Butterworth bandpass filter.

For the experiment, in the initial posture, the subjects were required to stand upright for 5 s with their weight equally distributed on both feet. Afterwards, the subjects were asked to walk for 1 min with self-selected gait speed, then stopped and waited for 5 s. A 5 min rest was given for each experiment interval to avoid potential muscle fatigue.

Common oscillatory drive to a muscle pair was quantified by EMG-EMG coherence [20], which was calculated using Welch's periodogram method with a Hamming window of 2048 samples and overlap of 1024 samples [20, 21]. The coherence was calculated using the following well-known equation [20, 22]:

(1) $$C_{ab}\left(\,f \right)= \displaystyle{{\left\vert {\left. {S_{ab}\left(\,f \right)} \right\vert } \right.} \over {S_{aa}\left(\,f \right)\ast S_{bb}\left(\,f \right)}}\eqno\lpar 1\rpar $$

where $S_{ab}\left(\,f \right)$ is cross-spectra and $S_{aa}\left(\,f \right)$, $S_{bb}\left(\,f \right)$ are the auto-spectra of *a*(*t*) and *b*(*t*) which are the EMG signals from one muscle pair at a time (RF-VL, RF-VM and RF-ST). Coherence assigns a unit less real number between 0 and 1; larger values (closer to 1) that correspond to two signals are perfectly coherent or synchronised in the frequency domain; smaller values (closer to 0) indicate that the two EMG signals are not coherent. One-way analysis of variance (ANOVA) was performed to compare the coherence calculated for each of the three muscle pairs (RF-VL, RF-VM and RF-ST) using three different heel heights. The ANOVA was performed using the MATLAB and Statistics Toolbox Release 2012a (The MathWorks Inc., Massachusetts, USA). The statistical level of significance was fixed at *p* < 0.05 (95% confidence intervals).

## 3 Results

**Fig. 1:**
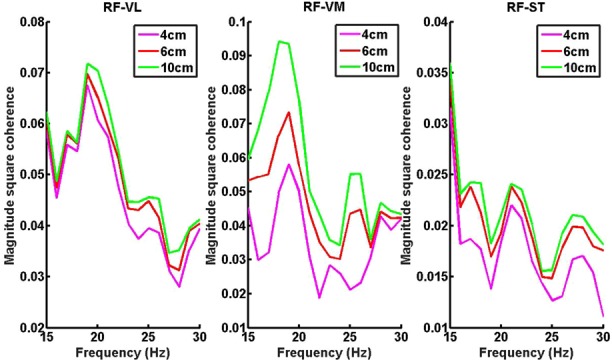
Sample beta band coherence result for one participant: beta band (15–30 Hz) coherence values for RF-VL, RF-VM and RF-ST muscle pairs, during self-paced walking on low (4 cm), medium (6 cm) and high (10 cm) heels shoes

**Fig. 2:**
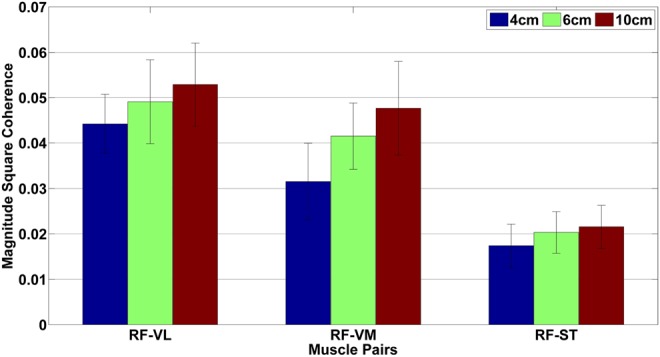
Average beta band (15–30 Hz) coherence values for RF-VL, RF-VM and RF-ST muscle pairs, during self-paced walking on low (4 cm), medium (6 cm) and high (10 cm) heels shoes

**Fig. 3:**
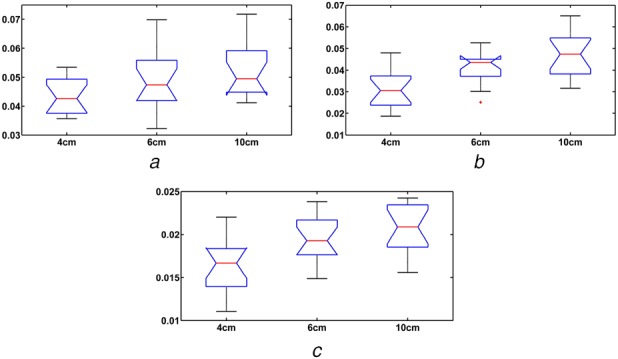
ANOVA results for *a* RF-VL muscle pairs *b* RF-VM muscle pairs *c* RF-ST muscle pairs, during self-paced walking on low (4 cm), medium (6 cm) and high (10 cm) heels shoes

Fig. 1 shows beta band coherence analysis of all three-muscle pairs (RF-VL, RF-VM and RF-ST) for one of the participant. The mean and standard deviation of beta band coherence values of all muscle pairs for different HHS (during walking) are given in Table 1. Average beta band (15–30 Hz) coherence values for RF-VL, RF-VM and RF-ST muscle pairs, during walking on low, medium and high heels shoes is shown in Fig. 2. The ANOVA results for RF-VL, RF-VM and RF-ST are shown in Fig. 3.

**Table 1:** Average beta band (15–30 Hz) coherence values of all muscle pairs of all 15 participants for different HHS during walking

Muscle pair	Low heel (4 cm)	Medium heel (6 cm)	High heel (10 cm)
RF-VL	0.0435 ± 0.0060^a^	0.0490 ± 0.0096^a^	0.0523 ± 0.0091^a^
RF-VM	0.0313 ± 0.0087^a^	0.0411 ± 0.0074^a^	0.0468 ± 0.0101^a^
RF-ST	0.0164 ± 0.0030^a^	0.0194 ± 0.0028^a^	0.0206 ± 0.0029^a^

^a^Significant at *p*<0.05.

From the results, it can be seen that beta band coherence value was significantly increased (*p*<0.05) during walking in RF-VL, RF-VM and RF-ST muscle pairs for all HHS.

## 4 Discussion

This study aimed to investigate at lower limb muscle synchronisation changes during walking on HHS. Previous studies found some kinematic modifications in knee movement while walking on HHS, which involves changes in lower limb muscle activities [7]. If there is any change in muscle activities, then change in muscle pair synchronisation must exist [16, 23], which is not well known yet for walking in HHS. During walking, quadriceps and hamstring muscles are mainly responsible for maintaining balance around the knee [9]. EMG-EMG coherence is a good measure to analyse this muscle synchronisation. According to previous research, higher beta band coherence was found in fatigue conditions [10, 20]. In this study, results showed significantly higher beta band coherence during walking in HHS for all muscle pairs, which indicates a higher level of muscle pair synchronisation is needed to walk on heels to maintain balance.

## 5 Conclusion

From this study, it can be concluded that walking in HHS increases beta band coherence and requires more muscle synchronisation to avoid imbalance around the knee as these muscles work for maintaining balance around knee by compensating their activation pattern together [9].

To conclude, we hypothesise that to maintain stability around the knee, the following physiological events occur:

- The lower limb muscles make short-term plastic changes in the neural drive while walking on HHS. Plasticity is defined as the ability of motor neurons and their respective effector muscles to change physically and functionally as a result of environmental conditions, activity, age and other factors [24].
- Higher coherence value reflects an adjusted neural strategy of the nervous system to control muscles during high-heeled walking. The most likely functional role of muscle synchronisation such as to perform the movement, to maintain posture is adjusted, by motor unit synchronisation. Increased coherence value or increased muscle synchronisation will actualise as an increase in the rate of force development during rapid contractions or a mechanism to coordinate the activity of multiple muscles [23].
